# Subjective Changes in Tobacco Product Use among Korean Adults during the COVID-19 Pandemic

**DOI:** 10.3390/ijerph19063272

**Published:** 2022-03-10

**Authors:** Jieun Hwang

**Affiliations:** College of Health Science, Dankook University, 119, Dandae-ro, Dongnam-gu, Cheonan-si 31116, Chungnam, Korea; hwang0310@dankook.ac.kr or loshjeve@gmail.com

**Keywords:** smoking, e-cigarette, heated tobacco product, COVID-19

## Abstract

This study explored subjective changes in tobacco product use during the COVID-19 pandemic. The online survey included 828 tobacco product users and was implemented from 20 August to 27 August 2021. Participants were classified based on currently used products (cigarettes, heated tobacco products (HTPs), and e-cigarettes) and categorized as single, dual, or triple users. Subjective changes in product use over the past year were designated as “no chang”, “increase”, or “decreased”. Single use was most common, as reported by 447 participants (male 249, female 198), while 283 (male 164, female 119) and 98 (male 59, female 39) participants reported dual and triple use, respectively. Age, income, and triple use were associated with increased cigarette use, whereas living with family was associated with decreased use. Gender, age, income, and triple use were associated with the changed use of HTPs. No factors were significantly associated with an increase in e-cigarette use, whereas age and income were associated with decreased use. Similar to many COVID-19-related changes in cultural, social, and economic aspects of life, users’ patterns of tobacco product use have also changed. Increasing tobacco product taxes, effective messaging, and customized cessation services might help prevent or limit tobacco product usage.

## 1. Introduction

The onset of smoking depends on several factors, including gender, age, race, socioeconomic status (SES), and puberty [1]. Smoking prevalence increases with increasing age, lower SES, and lower education level, and in males—compared to females—of Caucasian and Hispanic backgrounds [1]. In addition to these individual factors, genetic factors [2], as well as sociocultural factors such as region, have major influences on the onset of smoking [1].

After beginning to smoke, smokers justify their addiction by considering smoking to be a mode of relaxation and enjoyment [3]; however, they continue to smoke because the habit is founded upon nicotine addiction [4]. Persistent smoking is a major cause of death by cancer, cardiovascular disease, and respiratory disease [5]; these conditions adversely affect health and place a huge socioeconomic burden on the nation, as they are associated with a decrease in productivity due to premature death [6]. For this reason, many countries have implemented various tobacco control measures, such as raising taxes, expanding no-smoking zones, and placing health warning graphics on cigarette packs, in addition to promotion, marketing, and education aimed at preventing smoking and inducing smokers to quit [7].

Nevertheless, tobacco use is still prevalent among people. As of 2019, 32.7% of men and 6.6% of women in the global population (aged 15 and above) were smokers [8]. Cigarette use has decreased considerably. However, the use of other tobacco products, such as heated tobacco products (HTPs) and e-cigarettes, has diversified, and recently, their use has been spreading among adolescents and young adults [9].

Coronavirus disease 2019 (COVID-19) began spreading beyond China in January 2020 and has spread to most countries and all the continents, with a large number of cases of infections and deaths having been recorded since then [10]. Consequently, the World Health Organization (WHO) declared an international public health emergency on 31 January 2020, raised the global risk of COVID-19 to “very hig”. on 28 February, and declared it a pandemic on 11 March [11].

The pandemic affected health practices—there were health-related changes, such as a shift in the consumption of alcohol during the outbreak [12]. It was found that physical activities decreased with increases in sedentary time, whereas food intake and meal patterns became unhealthy [13]. Furthermore, the pandemic led to mental health problems, such as stress, anxiety, depressive symptoms, insomnia, denial, anger, and fear [14].

With the pandemic becoming a stress inducer, smokers may have increased the frequency of smoking to relieve stress [15]. Additionally, several studies have suggested that smokers are at a higher risk of contracting COVID-19 than healthy individuals and have poorer prognoses and outcomes [16]. Hence, the WHO has recommended discontinuing the use of tobacco products after the pandemic [17].

The changes in smoking habits have been diverse since the COVID-19 pandemic. A few studies reported that some people started smoking at the onset of the pandemic, some have increased the frequency of smoking [18,19,20,21], and even former smokers considered or started smoking again [22]. Meanwhile, some studies reported that the intent and attempts to quit smoking increased and the amount of smoking decreased [22].

However, the number of studies that have specifically examined the changes in different product uses since the COVID-19 pandemic is limited [18,19,20,21,22]. The changes in tobacco product use since the pandemic have been assessed; however, the effects of these changes, in terms of each product group, have not been investigated. Considering that various tobacco products are currently in use, it is necessary to investigate the changes associated with various products when conducting research.

Therefore, this study examined the changes in tobacco product usage within the past year among users to identify the underlying factors. The changes in the product groups were identified, and their similarities and differences were determined to provide directions for future tobacco control policies based on an understanding of smokers.

## 2. Materials and Methods

### 2.1. Data Source

Quota sampling was used in this study. As of June 2020, the total number of adults in Korea was 50,133,493 (men: 24,972,588; women: 25,160,905). The study population comprised 9,214,885 men (36.9%) and 1,559,976 women (6.2%), based on the current rate of tobacco product use. An online survey was conducted for seven days from 20 August to 27 August 2021. The participants were adults living in South Korea, recruited through Panel Marketing Interactive, a survey company. After explaining the purpose of the survey via e-mail, only those who voluntarily consented were allowed to participate. The entire survey took approximately 30 min, and the survey company made payments to each panelist who participated.

### 2.2. Measurements

Using quota sampling, a total of 828 participants were recruited based on gender, age, and region ratios. It is the most sophisticated method among non-probability sampling methods and has the advantage of generating a relatively representative sample. Our researchers tried not to bias the recruited panel by considering gender, age, and region according to each tobacco product use group. Current tobacco product users were determined by asking each person whether they currently use tobacco products. Based on the currently used product, the participants were divided into smokers, HTP users, and e-cigarette users. Smokers included those who had smoked more than 100 cigarettes within their lifetime and smoked cigarettes occasionally or daily. HTP users included those who smoked HTP products, such as IQOS, Lil, and Glo, occasionally or daily. E-cigarette users included those who smoked e-cigarette products occasionally or daily. In addition, depending on the product currently used, single use referred to using cigarettes, HTPs, or e-cigarettes; dual use referred to using cigarettes and HTPs, cigarettes and e-cigarettes, or HTPs and e-cigarettes; and triple use referred to using cigarettes, HTPs, and e-cigarettes. Subjective changes in product use over the past year were measured according to the products currently used. Current tobacco product users were asked “Has your cigarette/HTP/e-cigarette consumption changed over the past year”. According to their answers, the subjective change in tobacco product use was designated as increased, decreased, or no change.

In addition to the above, gender (male, female), age group (20–29, 30–39, 40–49, 50–59, 60–69), region (metropolitan, non-metropolitan), level of education (high school graduate, college graduate, and above college graduate), monthly income (less than KRW 3 million, KRW 3–5 million, KRW 5–7 million, and more than KRW 7 million), occupation (manager, office worker, service/labor worker, and other), and living arrangement (living with family and living alone) were also surveyed.

### 2.3. Statistical Analysis

SPSS version 26 was used for the statistical analysis. The differences in the distributions of respondents’ characteristics and tobacco product usage, stratified by gender, were determined using a chi-square test. The differences in the change in usage across the product groups were also confirmed using a chi-squared test. Logistic regression and multinomial logistic regression analyses of the change in product usage for each product group were performed, and the odds ratio (OR), adjusted odds ratio (aOR), and 95% confidence interval (CI) were tested at a significance level of 0.05.

## 3. Results

### 3.1. General Characteristics and Tobacco Use of Respondents

The age distribution of the 828 current tobacco users (472 males, 356 females) was as follows: 20–29 (19.5% males; 18.5% females), 30–39 (20.3% males; 20.8% females), 40–49 (24.8% males; 23.3% females), 50–59 (21.4% males; 22.5% females), and 60–69 (14.0% males; 14.9% females; Table 1). The difference among the five groups was not statistically significant (*p* = 0.971). The proportions of residents in the metropolitan (48.7% males; 50.8% females) and non-metropolitan areas (51.3% males; 49.2% females) were similar (*p* = 0.547). The proportion of college graduates was high among both males (73.5%) and females (70.2%), but females had a higher proportion of high school graduates than males (17.6% of males; 25.3% of females, *p* = 0.003). The proportion of females earning less than KRW 3 million was higher (19.3% of males; 27.2% of females), whereas the proportion of females earning KRW 3–5 million was lower, than that of males (38.3% of males; 30.1% of females), and this difference was statistically significant (*p* = 0.02). The proportion of office workers was high among both males and females (51.9% of males; 48.3% of females), but males had a higher proportion of managers than females (10.8% of males; 6.2% of females, *p* =< 0.001). Both males and females were more likely to live with family than alone, but the gender-related difference was not statistically significant (*p* = 0.522).

Among current tobacco product users, single use of cigarettes was the most common: 208 males (44.1%) and 151 females (42.4%) reported single use. This was followed by the combined use of cigarettes and HTPs among both males and females (27.3% of males, 23.9% of females); combined use of cigarettes, HTPs, and e-cigarettes (12.5% of males, 11% of females); combined use of cigarettes and e-cigarettes among males (7.0%); and HTP use among females (8.1%).

### 3.2. Change in Usage Stratified by Tobacco Product

Table 2 shows subjective changes in the use of tobacco products over the past year. Among males, cigarette use was shown in the order of no change (46.9%), increased (28.4%), and decreased (24.7%) (*p* = 0.001). Whereas cigarette use did not change among the majority of single users of cigarettes (51.4%) and dual users of cigarettes + e-cigarettes (46.3%), most triple users reported an increase (50.8%) in the use of cigarettes. For current HTP users, the use of HTPs was shown in the order of no change (46.4%), increased (32.0%), and decreased (21.6%) (*p* = 0.142). Most single users of HTPs (56.3%) and dual users (HTP + cigarettes or e-cigarettes) (48.1%) reported no change in their usage, whereas most triple users reported that their use of HTPs had increased (44.1%). Over the past year, the change in e-cigarette usage was shown in the order of increased (39.8%), no change (33.0%), and decreased (27.2%) (*p* = 0.923). The change in e-cigarette usage showed the same frequencies for increased, decreased, and no change for single users. Conversely, double (37.1%) and triple (42.4%) users showed the highest frequency for increased use.

Among females, cigarette use was shown in the order of no change (41.9%), decreased (30.0%), and increased (28.1%) (*p* = 0.001); however, most single (43.0%) and dual users (44.2%) reported no change in cigarette usage, whereas most triple users reported an increase (56.4%). As with cigarette use, HTP use was also reported in the order of no change (36.5%), decreased (34.6%), and increased (28.9%) (*p* = 0.143). Single users of HTPs most frequently reported no change (44.8%); dual users similarly reported no change (38.5%) and a decrease (38.5%) in HTP use; and triple users most frequently reported an increase (43.6%) in the use of HTPs. The use of e-cigarettes over a year decreased (37.4%), did not change (34.1%), and increased (28.6%) (*p* = 0.53). More than half of e-cigarette single users reported a decrease in usage (50.0%), whereas the dual (38.2%) and triple (38.5%) users predominantly reported no change in e-cigarette use.

### 3.3. Factors Affecting the Change in Usage of Tobacco Products

The factors that had a statistically significant association with an increase in cigarette use over the past year were age, income, and triple usage (Table 3). The younger participants had a higher likelihood of increased smoking (20–29 aOR = 3.69 (95% CI = 1.70–8.03); 30–39 aOR = 3.44 (95% CI = 1.59–7.44); 40–49 aOR = 2.51 (95% CI = 1.20–5.29); 50–59 aOR = 2.60 (95% CI = 1.23–5.49)), had a lower income (less than KRW 3 million aOR = 2.05 (95% CI = 1.07–3.95)), and were more likely to combine tobacco products instead of using only one (triple use aOR = 2.92 (95% CI = 1.67–5.08)). Meanwhile, living with family was associated with a decrease in cigarette use. It was more likely for cigarette users living with family to reduce cigarette use compared with those living alone (living with family aOR = 1.63 (95% CI = 1.09–2.42)).

The factors associated with an increase in HTP usage over the past year were age and the combined use of three different products (Table 4). The increase in usage in people in their 30s (aOR = 4.18 (95% CI = 1.47–11.86)) was greater than that observed in people in their 60s, followed by those in their 40s (aOR = 3.23 (95% CI = 1.14–9.18)). Compared with single users, triple users were 3.31 times (aOR = 3.31 (95% CI 1.48–7.40)) more likely to increase HTP use, whereas the factors associated with a decrease in HTP use were gender and income. In other words, compared with males, females (aOR = 0.44 (95% CI = 0.26–0.76)) were less likely to reduce their HTP use, and the probability of decreasing usage was higher for median-income earners (KRW 3–5 million aOR = 2.43 (95% CI = 1.08–5.47)) than for higher-income earners (KRW 7 million or higher).

No factors were associated with an increase in e-cigarette use over the past year (Table 5), while factors associated with a decrease in e-cigarette use were age and income. In other words, a reduction in the use of e-cigarettes was less likely for younger users (30–39 aOR = 0.07 (95% CI = 0.01–0.45); 40–49 aOR = 0.09 (95% CI = 0.01–0.63)), and more likely for low-income earners (less than KRW 3 million aOR = 4.49 (95% CI = 1.13–17.83); KRW 3–5 million aOR = 3.57 (95% CI = 1.10–11.53)).

## 4. Discussion

### 4.1. General Discussion

This study confirmed that Korean smokers use various tobacco products. The findings also show that the range of tobacco products used by tobacco users is becoming more diverse than in previous studies. A study conducted in South Korea in September 2017 showed that all HTP users use both cigarettes and e-cigarettes [23], whereas a study conducted in December 2018, among 1530 tobacco users, showed that 12.7% of the participants were triple users [24], similar to the figure reported in this study (11.8%).

E-cigarettes have been selling in South Korea since 2007, and HTPs since 2017. Since then, even though cigarettes have recorded the highest sales within the category, the sales and use of other tobacco products are on the rise [25]. According to a national survey, the rate of cigarette usage was 45.1% among males and 5.3% among females in 2007, and 35.7% among males and 6.7% among females in 2019, whereas e-cigarette use increased from 2.0% among males and 0.3% among females in 2013 to 5.1% among males and 1.4% among females in 2019. Additionally, the rate of HTP use was 7.4% among males and 0.9% among females as of 2018. Compared with the results of a survey conducted in December 2018 [24], the present findings show that the number of single users of HTPs decreased, but the single and double users of HTPs showed an increasing trend, indicating increased use of HTPs.

This study was conducted during the COVID-19 pandemic, and the subjective change in respondents’ use of tobacco products over the year was assessed. We found that although there were changes in tobacco use during this period, they do not show any encouraging reduction in such usage. This is despite the fact that several studies have been conducted on the association of smoking with COVID-19, and various countries have attempted to induce smoking cessation by citing smoking as a risk factor [16]. A study in China showed that 30.1% of smokers increased their tobacco use, whereas 69.1% did not increase smoking during the pandemic [19], and a United Kingdom study showed that 50.9% of current smokers reported smoking the same amount as before [20]. Given that tobacco products are mainly used out of habit or addiction [26], it would be difficult to reduce such use even with the prevalence of large-scale infectious diseases, such as COVID-19.

Despite the warning that smoking is a risk factor for diseases such as COVID-19, there have been cases where people’s smoking behavior did not change or even increased. It is not easy for smokers to reduce or quit smoking even under such critical circumstances. Thus, prevention is the most important way forward. However, our research results indicate the factors that influence the reduction in tobacco product use. First, the use of cigarettes was significantly reduced by those living with family. This could be attributed to the dangers associated with tobacco products, posed against family members, being emphasized, which led to a behavioral change [27]. Specifically, the increase in the time spent at home during the pandemic may have influenced the reduction in cigarette use. These results necessitate the development of messages that effectively communicate the dangers of second-hand/third-hand smoke in homes and emphasize that smoking affects not only the user’s health but also the health of their closest family members.

The “incom”. factor was associated with a decrease in the use of HTPs and e-cigarettes, suggesting that the cost of products may have had a significant effect. Some studies have reported the cost of products as the main reason for choosing cigarettes [28]; the cost of HTPs and e-cigarettes may have been a burden for actual users. Cost affects the approach, initiation, and maintenance of a product—a product may be initially used out of curiosity, but its cost determines whether its use will be maintained [29]. Considering that the nicotine content of HTPs and e-cigarettes may be lower than that of cigarettes [30], more frequent use is required for ingestion of a satisfactory amount of nicotine, which is not an advantage when it comes to its continued use. In particular, it is necessary to pay attention to the change in usage of triple users. Although the use of e-cigarettes among triple users has decreased, the use of regular cigarettes and HTPs has increased.

In addition to the above, the increase in the use of products among triple users was notable. Although the use of e-cigarettes among triple users decreased, the use of cigarettes and HTPs increased. This finding is contrary to the opinion that using various products helps in smoking cessation, by reducing the use of cigarettes [31]; it even seems that using various products makes the smokers continue smoking. Different tobacco products could be used regardless of location and time, and smoking becomes easier. These results suggest that the public should be fully informed that combined tobacco use may pose physical health risks through continued exposure to nicotine [32].

Additionally, the combined use of products was more likely among younger participants. The likelihood of increasing cigarette use was highest among individuals in their 20s, followed by those in their 30s, 50s, and 40s; for increasing HTP use, the likelihood was the highest among those in their 30s, followed by those in their 40s and 20s. Based on these results, smoking may have increased due to stress among participants in the age groups that were more likely to have been affected by the social changes after COVID-19. Younger people tend to be more insecure regarding education and work, though it is also highly likely that factors caused by the COVID-19 pandemic that adversely affected young people, such as education, employment, and their future, acted as stressors [33]. These results emphasize the need to integrate mental health treatment not only in primary care but also in overall health care [34].

The results of this study are relevant, as it is the first to assess subjective changes in tobacco use after COVID-19 in South Korea. However, this research has the following limitations. First, caution is required in interpreting the results, which may have been overestimated because the participants were recruited using assigned sampling for each tobacco product, and there was a larger proportion of HTP and e-cigarette users than in the general population. Additionally, the study assessed the subjective changes in product use among tobacco product users over a year, without investigating changes in economic status, mental health status, and other health behaviors, making it difficult to attribute the changes only to COVID-19. Finally, other factors that affect the use of tobacco products, such as alcohol use and physical activity, were not examined, and a more detailed survey incorporating these factors is required.

### 4.2. Implications

Similar to the many COVID-19-related changes in cultural, social, and economic aspects of life, users’ patterns of tobacco product use have also changed. Compared to single users, cigarette and HTP use by triple users has increased over the past year, suggesting that product diversification allows tobacco users to continue their use without interruption and that they cannot escape from nicotine addiction despite the pandemic. Therefore, future tobacco-related studies should consider various tobacco products. Accordingly, tobacco control policy should focus on the prevention of initiation above all else. A multi-faceted approach to free tobacco users from nicotine addiction is an urgent necessity.

## 5. Conclusions

This study assessed the subjective changes in tobacco product use among current users in South Korea over a year after the COVID-19 outbreak, and the factors underlying such use. Most tobacco product users were single users, but significant proportions of them were dual and triple users. Most users changed their tobacco use behavior in terms of either an increase or decrease in their product use. Age, income, and triple use were associated with increased cigarette use, whereas living with family was associated with decreased use. Gender, age, income, and triple use were associated with the changed use of HTPs. No factors were significantly associated with the increase in e-cigarette use, whereas age and income were associated with decreased use. The results of this study show that there were no change and, in some cases, an increase in usage by smokers. Therefore, smoking cessation services should be provided in various ways to induce reduction and quitting, and strategic promotional messages to protect the health of others, such as family and neighbors, should be disseminated. Additionally, a strategic promotion targeted at current users is required to protect the health of families and others.

## Figures and Tables

**Table 1:** Characteristics of the participants (n = 828).

Variables		Male (n = 472)		Female (n = 356)		*p*-Value ^†^
		n	%	n	%	
Age group						0.971
	20–29	92	19.5	66	18.5	
	30–39	96	20.3	74	20.8	
	40–49	117	24.8	83	23.3	
	50–59	101	21.4	80	22.5	
	60–69	66	14.0	53	14.9	
Region						0.547
	Metropolitan	230	48.7	181	50.8	
	Non-metropolitan	242	51.3	175	49.2	
Education						0.003
	Below high school graduate	83	17.6	90	25.3	
	College graduate	347	73.5	250	70.2	
	Higher than college graduate	42	8.9	16	4.5	
Monthly income						0.020
	Less than KRW 3 million	91	19.3	97	27.2	
	KRW 3 to 5 million	181	38.3	107	30.1	
	KRW 5 to 7 million	115	24.4	91	25.6	
	KRW 7 million or higher	85	18.0	61	17.1	
Occupation						<0.001
	Manager	51	10.8	22	6.2	
	Office worker	245	51.9	172	48.3	
	Service/Labor worker	114	24.2	77	21.6	
	Other	62	13.1	85	23.9	
Living arrangement						0.522
	Living with family	298	63.1	217	61.0	
	Living alone	174	36.9	139	39.0	
Current tobacco product use						0.069
Single users		249	52.8	198	55.6	
	Smoker (cigarettes)	208	44.1	151	42.4	
	HTPs	32	6.8	29	8.1	
	E-cigarettes	9	1.9	18	5.1	
Dual users		164	34.7	119	33.4	
	Cigarettes + HTPs	129	27.3	85	23.9	
	Cigarettes + e-cigarettes	33	7.0	28	7.9	
	HTPs + e-cigarettes	2	0.4	6	1.7	
Triple users						
	Cigarettes + HTPs + e-cigarettes	59	12.5	39	11.0	

^†^ Chi-square test.

**Table 2:** Subjective changes in usage stratified by tobacco product over the past year.

		Increased		Decreased		No Change		*p*-Value ^†^
		n	%	n	%	n	%	
Male	Use of cigarettes (n = 429)	122	28.4	106	24.7	201	46.9	0.001
	single user (n = 208)	46	22.1	55	26.4	107	51.4	
	dual user (n = 162)	46	28.4	41	25.3	75	46.3	
	triple user (n = 59)	30	50.8	10	16.9	19	32.2	
	Use of HTPs (n = 222)	71	32.0	48	21.6	103	46.4	0.142
	single user (n = 32)	6	18.8	8	25.0	18	56.3	
	dual user (n = 131)	39	29.8	29	22.1	63	48.1	
	triple user (n = 59)	26	44.1	11	18.6	22	37.3	
	Use of e-cigarettes (n = 103)	41	39.8	28	27.2	34	33.0	0.923
	single user (n = 9)	3	33.3	3	33.3	3	33.3	
	dual user (n = 35)	13	37.1	11	31.4	11	31.4	
	triple user (n = 59)	25	42.4	14	23.7	20	33.9	
Female	Use of cigarettes (n = 303)	85	28.1	91	30.0	127	41.9	0.001
	single user (n = 151)	35	23.2	51	33.8	65	43.0	
	dual user (n = 113)	28	24.8	35	31.0	50	44.2	
	triple user (n = 39)	22	56.4	5	12.8	12	30.8	
	Use of HTPs (n = 159)	46	28.9	55	34.6	58	36.5	0.143
	single user (n = 29)	8	27.6	8	27.6	13	44.8	
	dual user (n = 91)	21	23.1	35	38.5	35	38.5	
	triple user (n = 39)	17	43.6	12	30.8	10	25.6	
	Use of e-cigarettes (n = 91)	26	28.6	34	37.4	31	34.1	0.53
	single user (n = 18)	6	33.3	9	50.0	3	16.7	
	dual user (n = 34)	9	26.5	12	35.3	13	38.2	
	triple user (n = 39)	11	28.2	13	33.3	15	38.5	

^†^ Chi-square test.

**Table 3:** Factors associated with the subjective change in usage of cigarettes.

Variables		Cigarettes											
		Increased						Decreased					
		OR	(95% CI)	*p*-Value	aOR	(95% CI)	*p*-Value	OR	(95% CI)	*p*-Value	aOR	(95% CI)	*p*-Value
Gender													
	Male	0.91	(0.64–1.29)	0.59	0.90	(0.62–1.32)	0.60	0.74	(0.52–1.05)	0.09	0.72	(0.50–1.04)	0.08
	Female	ref.			ref.			ref.			ref.		
Age group													
	20–29	**4.44**	**(2.14–9.24)**	**<0.001**	**3.69**	**(1.70–8.03)**	**<0.001**	0.92	(0.51–1.65)	0.77	1.08	(0.58–2.03)	0.81
	30–39	**4.47**	**(2.17–9.25)**	**<0.001**	**3.44**	**(1.59–7.44)**	**<0.001**	0.79	(0.44–1.43)	0.44	0.79	(0.42–1.50)	0.47
	40–49	**3.21**	**(1.58–6.54)**	**<0.001**	**2.51**	**(1.20–5.29)**	**0.02**	0.83	(0.49–1.43)	0.51	0.76	(0.43–1.35)	0.35
	50–59	**2.90**	**(1.41–5.96)**	**<0.001**	**2.60**	**(1.23–5.49)**	**0.01**	0.97	(0.57–1.66)	0.90	0.96	(0.55–1.69)	0.90
	60–69	ref.			ref.			ref.			ref.		
Region													
	Metropolitan	1.17	(0.82–1.65)	0.39	1.05	(0.72–1.53)	0.80	0.95	(0.67–1.36)	0.80	1.01	(0.70–1.47)	0.94
	Non-metropolitan	ref.			ref.			ref.			ref.		
Education													
	Below high school graduate	0.92	(0.42–2.01)	0.83	0.74	(0.30–1.82)	0.51	0.97	(0.44–2.10)	0.93	0.64	(0.27–1.53)	0.32
	College graduate	1.20	(0.59–2.43)	0.62	0.81	(0.37–1.76)	0.59	1.10	(0.54–2.25)	0.79	0.95	(0.45–2.03)	0.90
	Above college graduate	ref.			ref.			ref.			ref.		
Monthly income													
	Less than KRW 3 million	**2.05**	**(1.15–3.65)**	**0.02**	**2.05**	**(1.07–3.95)**	**0.03**	1.25	(0.72–2.17)	0.43	1.36	(0.74–2.52)	0.33
	KRW 3 to 5 million	1.27	(0.73–2.20)	0.39	1.14	(0.63–2.05)	0.67	1.08	(0.66–1.77)	0.76	1.10	(0.65–1.87)	0.73
	KRW 5 to 7 million	**2.17**	**(1.23–3.81)**	**0.01**	**1.93**	**(1.06–3.51)**	**0.03**	**0.89**	**(0.51–1.56)**	**0.68**	0.93	(0.52–1.69)	0.82
	KRW 7 million or higher	ref.			ref.			ref.			ref.		
Occupation													
	Manager	0.77	(0.36–1.66)	0.50	0.79	(0.34–1.87)	0.60	0.82	(0.41–1.64)	0.57	0.89	(0.41–1.91)	0.76
	Office worker	1.55	(0.09–2.55)	0.08	1.26	(0.71–2.23)	0.43	0.87	(0.54–1.41)	0.57	0.96	(0.56–1.65)	0.89
	Service/Labor worker	1.29	(0.73–2.27)	0.39	1.32	(0.72–2.43)	0.37	1.16	(0.68–1.98)	0.59	1.36	(0.78–2.38)	0.28
	Other	ref.			ref.			ref.			ref.		
Living arrangement													
	Living with family	1.09	(0.76–1.56)	0.63	1.22	(0.82–1.81)	0.33	**1.50**	**(1.03–2.18)**	**0.03**	**1.63**	**(1.09–2.42)**	**0.02**
	Living alone	ref.			ref.						ref.		
Tobacco product													
	Triple use	**3.56**	**(2.12–5.98)**	**<0.001**	**2.92**	**(1.67–5.08)**	**0.00**	0.79	(0.41–1.52)	0.47	0.77	(0.39–1.53)	0.46
	Dual use	1.26	(0.85–1.86)	0.25	1.22	(0.81–1.84)	0.34	0.99	(0.68–1.43)	0.94	0.96	(0.65–1.42)	0.86
	Single use	ref.			ref.			ref.			ref.		

OR: odds ratio; aOR: adjusted odds ratio; CI: confidence interval. Adjusted odds ratios (aORs) are adjusted for all variables shown. The significant *p*-values (*p* < 0.05) are in bold.

**Table 4:** Factors associated with the change in usage of heated tobacco products.

Variables		Heated Tobacco Products											
		Increased						Decreased					
		OR	(95% CI)	*p*-Value	aOR	(95% CI)	*p*-Value	OR	(95% CI)	*p*-Value	aOR	(95% CI)	*p*-Value
Gender													
	Male	0.87	(0.53–1.42)	0.58	0.78	(0.46–1.32)	0.35	**0.49**	**(0.30–0.81)**	**0.01**	**0.44**	**(0.26–0.76)**	**0.00**
	Female	ref.			ref.			ref.			ref.		
Age group													
	20–29	2.44	(0.92–6.46)	0.07	2.13	(0.74–6.12)	**0.16**	0.80	(0.36–1.76)	0.58	1.01	(0.41–2.50)	0.98
	30–39	**4.20**	**(1.60–11.05)**	**<0.001**	**4.18**	**(1.47–11.86)**	**0.01**	0.73	(0.32–1.69)	0.47	0.85	(0.33–2.16)	0.73
	40–49	**2.95**	**(1.12–7.78)**	**0.03**	**3.23**	**(1.14–9.18)**	**0.03**	0.84	(0.38–1.86)	0.67	0.93	(0.38–2.28)	0.88
	50–59	1.50	(0.53–4.29)	0.45	1.51	(0.51–4.49)	0.46	0.71	(0.31–1.64)	0.42	0.77	(0.32–1.90)	0.58
	60–69	ref.			ref.			ref.			ref.		
Region													
	Metropolitan	1.19	(0.74–1.93)	0.47	1.03	(0.61–1.73)	0.93	0.67	(0.41–1.10)	0.11	0.69	(0.40–1.18)	0.18
	Non-metropolitan	ref.			ref.			ref.			ref.		
Education													
	Below high school graduate	0.66	(0.24–1.82)	0.42	0.52	(0.15–1.80)	0.30	0.89	(0.29–2.77)	0.85	0.42	(0.11–1.62)	0.21
	College graduate	0.89	(0.38–2.08)	0.79	0.61	(0.24–1.57)	0.31	1.27	(0.49–3.33)	0.62	0.94	(0.33–2.72)	0.91
	Above college graduate	ref.			ref.			ref.			ref.		
Monthly income													
	Less than KRW 3 million	1.19	(0.55–2.60)	0.66	0.90	(0.35–2.29)	0.82	1.75	(0.76–4.01)	0.19	2.09	(0.79–5.55)	0.14
	KRW 3 to 5 million	1.12	(0.57–2.20)	0.74	0.84	(0.39–1.78)	0.65	1.86	(0.90–3.84)	0.09	**2.43**	**(1.08–5.47)**	**0.03**
	KRW 5 to 7 million	1.30	(0.65–2.60)	0.46	1.00	(0.47–2.12)	1.00	1.27	(0.58–2.79)	0.55	1.43	(0.61–3.34)	0.41
	KRW 7 million or higher	ref.			ref.			ref.			ref.		
Occupation													
	Manager	0.77	(0.25–2.42)	0.65	0.55	(0.15–2.02)	0.37	0.84	(0.30–2.37)	0.74	1.23	(0.37–4.10)	0.74
	Office worker	1.24	(0.58–2.64)	0.58	0.84	(0.35–2.00)	0.69	0.67	(0.32–1.38)	0.27	0.62	(0.27–1.45)	0.27
	Service/Labor worker	0.97	(0.39–2.41)	0.96	0.83	(0.32–2.19)	0.71	0.82	(0.35–1.93)	0.66	0.81	(0.32–2.03)	0.66
	Other	ref.			ref.			ref.			ref.		
Living arrangement													
	Living with family	0.82	(0.50–1.33)	0.42	0.75	(0.42–1.32)	0.32	1.54	(0.89–2.66)	0.12	1.64	(0.88–3.03)	0.12
	Living alone	ref.			ref.			ref.			ref.		
Tobacco product													
	Triple use	**2.98**	**(1.37–6.49)**	**0.01**	**3.31**	**(1.48–7.40)**	**0.00**	1.39	(0.62–3.12)	0.42	1.39	(0.60–3.23)	0.45
	Dual use	1.36	(0.67–2.75)	0.40	1.78	(0.85–3.73)	0.13	1.27	(0.64–2.50)	0.50	1.29	(0.63–2.67)	0.49
	Single use	ref.			ref.			ref.			ref.		

OR: odds ratio; aOR: adjusted odds ratio; CI: confidence interval. Adjusted odds ratios (aORs) are adjusted for all variables shown. The significant *p*-values (*p* < 0.05) are in bold.

**Table 5:** Factors associated with the change in usage of e-cigarettes.

Variables		E-cigarettes											
		Increased						Decreased					
		OR	(95% CI)	*p*-Value	Aor	(95% CI)	*p*-Value	OR	(95% CI)	*p*-Value	Aor	(95% CI)	*p*-Value
Gender													
	Male	1.44	(0.72–2.87)	0.30	1.50	(0.67–3.33)	0.32	0.75	(0.37–1.51)	0.42	0.72	(0.31–1.65)	0.44
	Female	ref.			ref.			ref.			ref.		
Age group													
	20–29	2.13	(0.18–26.03)	0.55	1.41	(0.10–20.42)	0.80	0.30	(0.06–1.63)	0.16	0.17	(0.03–1.14)	0.07
	30–39	1.67	(0.14–19.76)	0.69	1.18	(0.09–16.06)	0.90	**0.14**	**(0.03–0.73)**	**0.02**	**0.07**	**(0.01–0.45)**	**0.01**
	40–49	2.67	(0.22–32.23)	0.44	2.08	(0.14–30.10)	0.59	**0.17**	**(0.03–0.95)**	**0.04**	**0.09**	**(0.01–0.63)**	**0.02**
	50–59	2.22	(0.17–28.86)	0.54	2.09	(0.14–30.87)	0.59	0.36	(0.06–2.11)	0.26	0.31	(0.05–2.09	0.23
	60–69	ref.			ref.			ref.			ref.		
Region													
	Metropolitan	0.87	(0.43–1.75)	0.70	0.94	(0.44–2.04)	0.88	0.63	(0.31–1.27)	0.19	0.58	(0.26–1.29)	0.18
	Non-metropolitan	ref.			ref.			ref.			ref.		
Education													
	Below high school graduate	0.33	(0.06–1.75)	0.19	0.20	(0.03–1.34)	0.10	1.25	(0.26–6.07)	0.78	0.85	(0.12–6.09)	0.88
	College graduate	0.95	(0.27–3.30)	0.94	0.69	(0.17–2.73)	0.60	1.20	(0.30–4.74)	0.80	1.49	(0.28–7.78)	0.64
	Above college graduate	ref.			ref.			ref.			ref.		
Monthly income													
	Less than KRW 3 million	2.00	(0.61–6.58)	0.25	3.57	(0.88–14.47)	0.07	2.18	(0.72–6.61)	0.17	**4.49**	**(1.13–17.83)**	**0.03**
	KRW 3 to 5 million	2.71	(0.94–7.77)	0.06	2.94	(0.93–9.27)	0.07	2.45	(0.90–6.65)	0.08	**3.57**	**(1.10–11.53)**	**0.03**
	KRW 5 to 7 million	2.38	(0.85–6.66)	0.10	2.48	(0.83–7.38)	0.10	0.84	(0.29–2.46)	0.75	1.12	(0.34–3.71)	0.85
	KRW 7 million or higher	ref.			ref.			ref.			ref.		
Occupation													
	Manager	0.29	(0.05–1.59)	0.15	0.31	(0.05–2.00)	0.22	0.29	(0.06–1.46)	0.13	0.34	(0.05–2.27)	0.27
	Office worker	0.56	(0.16–2.00)	0.37	0.49	(0.12–2.03)	0.33	0.32	(0.09–1.12)	0.08	0.35	(0.08–1.44)	0.15
	Service/Labor worker	0.35	(0.08–1.51)	0.16	0.26	(0.05–1.27)	0.10	0.43	(0.11–1.72)	0.23	0.24	(0.05–1.13)	0.07
	Other	ref.			ref.			ref.			ref.		
Living arrangement													
	Living with family	0.93	(0.46–1.86)	0.83	0.67	(0.29–1.51)	0.33	1.14	(0.55–2.34)	0.73	1.14	(0.48–2.69)	0.76
	Living alone	ref.			ref.			ref.			ref.		
Tobacco product													
	Triple use	0.69	(0.22–2.13)	0.51	0.54	(0.15–1.89)	0.33	0.39	(0.13–1.16)	0.09	0.34	(0.10–1.20)	0.09
	Dual use	0.61	(0.19–2.00)	0.42	0.57	(0.15–2.10)	0.40	0.48	(0.15–1.49)	0.20	0.46	(0.12–1.67)	0.24
	Single use	ref.			ref.			ref.			ref.		

OR: odds ratio; aOR: adjusted odds ratio; CI: confidence interval. Adjusted odds ratios (aORs) are adjusted for all variables shown. The significant *p*-values (*p* < 0.05) are in bold.

## Data Availability

Not available.
